# A Novel Methodology for Classifying EMG Movements Based on SVM and Genetic Algorithms

**DOI:** 10.3390/mi13122108

**Published:** 2022-11-29

**Authors:** Marcos Aviles, Luz-María Sánchez-Reyes, Rita Q. Fuentes-Aguilar, Diana C. Toledo-Pérez, Juvenal Rodríguez-Reséndiz

**Affiliations:** 1 Faculty of Engineering, Universidad Autónoma de Querétaro, Querétaro 76010, Mexico; 2 Tecnológico de Monterrey, Institute of Advanced Materials for Sustainable Manufacturing, Guadalajara 45201, Mexico

**Keywords:** support vector machine, metaheuristic algorithms, feature selection, electromyography, pattern recognition

## Abstract

Electromyography (EMG) processing is a fundamental part of medical research. It offers the possibility of developing new devices and techniques for the diagnosis, treatment, care, and rehabilitation of patients, in most cases non-invasively. However, EMG signals are random, non-stationary, and non-linear, making their classification difficult. Due to this, it is of vital importance to define which factors are helpful for the classification process. In order to improve this process, it is possible to apply algorithms capable of identifying which features are most important in the categorization process. Algorithms based on metaheuristic methods have demonstrated an ability to search for suitable subsets of features for optimization problems. Therefore, this work proposes a methodology based on genetic algorithms for feature selection to find the parameter space that offers the slightest classification error in 250 ms signal segments. For classification, a support vector machine is used. For this work, two databases were used, the first corresponding to the right upper extremity and the second formed by movements of the right lower extremity. For both databases, a feature space reduction of over 65% was obtained, with a higher average classification efficiency of 91% for the best subset of parameters. In addition, particle swarm optimization (PSO) was applied based on right upper extremity data, obtaining an 88% average error and a 46% reduction for the best subset of parameters. Finally, a sensitivity analysis was applied to the characteristics selected by PSO and genetic algorithms for the database of the right upper extremity, obtaining that the parameters determined by the genetic algorithms show greater sensitivity for the classification process.

## 1. Introduction

In recent years, myoelectric signal processing has been implemented in device control because using EMG signals from users provides a more intuitive system due to natural control. This has led to the development of devices with greater accessibility, more comfortable, and a greater degree of freedom. Despite this, the interpretation of muscle signals represents a significant challenge since they are immersed in a large amount of noise, which makes it difficult to classify them without prior processing [1]. This difficulty lies in that the electrical pulses generated by muscles are non-periodic, with non-stationary and low-voltage properties. That is due to the acquisition being performed superficially through the skin, and fat layers, which makes it difficult to read them [2,3].

Due to the above, machine learning algorithms focused on EMG recognition have preprocessing, data segmentation, parameter calculation, feature selection, and classification stages. One of the most relevant stages is parameter extraction, and the most critical features hidden within the acquired signals are obtained during this step. Subsequently, the best combination is selected to form a matrix of features that will be used in the classification stage. Finally, in the classification section, various algorithms are used, including neural networks, decision trees, linear discriminant analysis, and support vector machines (SVM), among others [4,5].

SVM is one of the most widely used EMG classification techniques because it has the potential to differentiate complex patterns due to its versatility and robustness with non-stationary data. Several works have been conducted where SVM is compared against other classifiers and has shown better classification accuracy. Some of the classifiers against which it has been compared are artificial neural networks, linear discriminant analysis, and particle swarm optimization [5,6,7,8,9].

A crucial issue is finding a feature set that offers the highest-ranking percentage and robustness to the system. The advantages that are obtained due to feature selection are the direct reduction of the size of the subset of features, acceleration of the training time, and simplification of the classifiers to improve the accuracy of the classification performance [10].

Metaheuristic algorithms are widely used to provide satisfactory solutions at high speed when dealing with complex spaces and areas in the industry, neural networks, image domain, signal classification, etc. [11,12]. Most of these algorithms are inspired by nature, and their goal is to find a global or minimum solution during the iterative search. The given solutions are perceived as more representative than studies focusing on stochastic methods. This promotes and encourages scholars to use metaheuristic algorithms to solve problems in different fields, such as EMG signal classification [13,14]. Genetic algorithms are one of the most famous exponents of this class of algorithms.

There is a need to create intuitive, efficient, and cost-effective systems as myoelectric control systems offer device manipulation alternatives. This work focuses on implementing genetic algorithms and SVM to classify muscle signals. The results indicate a substantial improvement in efficiency due to the parameter selection process, ranging from a 9% to a 36% reduction in the magnitude of the error, which is accompanied by a 65% decrease in the feature space.

The present work is divided as follows. In Section 2, a literature review is presented to provide an introspective of the proposed work. In Section 3, the steps to follow to develop the proposed algorithm are presented. The findings found are shown in Section 4. Finally, the scope areas are presented in Section 5 and Section 6.

## 2. Related Work

Table 1 compares the feature selection methods and the classifier used in various works. The most relevant projects for this work are described in detail below.

Cai et al. [15] presented a methodology to classify eight facial expressions for one person based on 12 features, eight in the time domain and four in the frequency domain. Six channels to carry out the classification were used: each channel has 12 features, and two are combined features of six channels, giving a total of 74 features. Additionally, windows of 2000 points are used, generating 30 repetitions for each expression. However, for this work, a selection method was not carried out. On the contrary, it was based on the literature to choose its parameters.

Too and Abdullah [16] propose an algorithm for classifying ten different hand and wrist movements, using windows of 200 ms and 33 characteristics corresponding to four channels, giving a total of 132 parameters. The database has eight people. However, the selection of characteristics, training, and validation of the classification algorithm was accomplished for individual subject giving eight classification percentages, achieving an average precision in the 94% rating.

Too et al. [17] worked on classifying 17 hand movements of 10 healthy people using 12 electrodes and eight frequency domain characteristics, giving a total of 120 parameters for classification. An average classification of 94% was achieved; the selection, training, and classification process were executed for individuals that made up the database.

**Table 1:** Comparative table of classification algorithms, selection methods, and classification accuracy.

Ref.	Feature Selection Method	Classification Algorithm	Classification Accuracy
[18]	ANOVA analysis	Radial basis SVM	91%
[15]	Literature review	Cubic SVM, Cubic KNN, and Gaussian SVM	99.52%, 97.98% and 94.94% respectively
[19]	Literature review	RBF SVM	94%
[20]	Literature review	Naive Bayes (NB) and SVM	73.6% and 77.6%
[21]	Particle swam optimization (PSO)	Radial basis SVM	90.6%
[22]	Literature review	Convolutional neural network and an SVM	-
[23]	Sequential forward selection (SFS) and PSO	Linear SVM	71%
[24]	A hybrid version of binary particle swarm optimization differential evolution	*k*-nearest neighbor (KNN)	92.5%
[16]	Opposition based competitive gray wolf optimizer	KNN	94% average
[17]	Competitive binary gray wolf optimizer	KNN	94.9% average
[8]	PSO and colony optimization	Linear discriminant analysis, KNN and SVM	Average LDA accuracy = 90%, average SVM accuracy = 92% and average SVM accuracy = 91%

It is noted that there are different methods of feature selection. However, several problems must be addressed. One of them is to include in the selection and classification of all the individuals that make up a database and not implement these methodological steps for each person individually, with the aim of developing a robust classification algorithm with a more significant potential for generalization. Finally, although genetic algorithms have been widely used in various areas, they have not been fully explored for feature selection for EMG signals, since they can be computationally expensive. Evaluating each individual requires training a model, and these algorithms can take a long time to converge, since they are stochastic.

In contrast, genetic algorithms perform better than traditional feature selection techniques. They can handle feature-rich data sets, they do not require specific knowledge about the problem under study, and these algorithms can be easily parallelized across computer clusters. Given the previous panorama, this work proposes a classification algorithm using genetic algorithms for selecting characteristics, encompassing all patients in the available databases using a low number of channels in the methodology.

## 3. Materials and Methods

The Materials and Methods section describes the different stages of developing the feature classification and selection system. It is important to note that the development of the selection and classification algorithm was implemented with the database presented in [25]. On the other hand, the validation and verification of the algorithm were accomplished with the database developed.

Preprocessing, characterization, feature selection, classification, and data analysis were made in *MATLAB R2018a*. These processes were carried out on a *Lenovo Legion Y520-15IKBN* laptop with 8GB of RAM and a 2.5 GHz Intel core i5-7300HQ processor.

### 3.1. EMG System

This work developed an EMG acquisition system to create the database. The board has a 3.5 mm headphone jack connected to the differential inputs. The instrumentation amplifier INA118UA was implemented to create each bipolar channel. Its maximum input value is 50 mV. An operational amplifier LMC6064 is connected in an integrating configuration in feedback between the output and the reference with a cutoff frequency of 1.6 Hz to eliminate or adjust the offset due to the skin–electrode interface at the input. The next stage consists of the filters.

The filtering part was designed using the Filter Design Tool from Texas Instruments and Multisim 14 to simulate the response of the circuits. In this stage, analog filtering was carried out from 10 to 500 Hz, using a low-pass and high-pass filter in series, both second-order Sallen–Key topologies. Finally, a second-order Bainter–Notch notch filter filters the 60 Hz signal from the power supply. The described filters were carried out using the LMC6064 operational amplifier. It should be noted that a buffer amplifier was placed at the input of each of the filters to match impedances between filter stages and avoid signal distortion [26].

**Figure 1:**
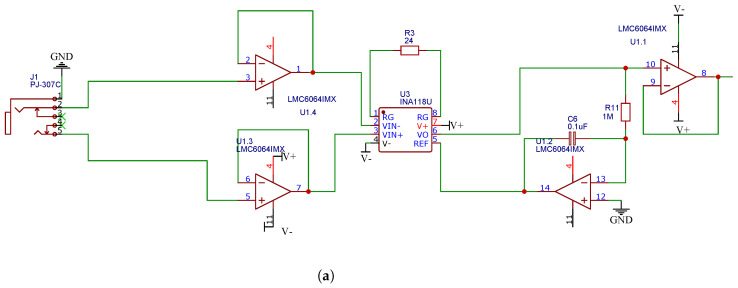

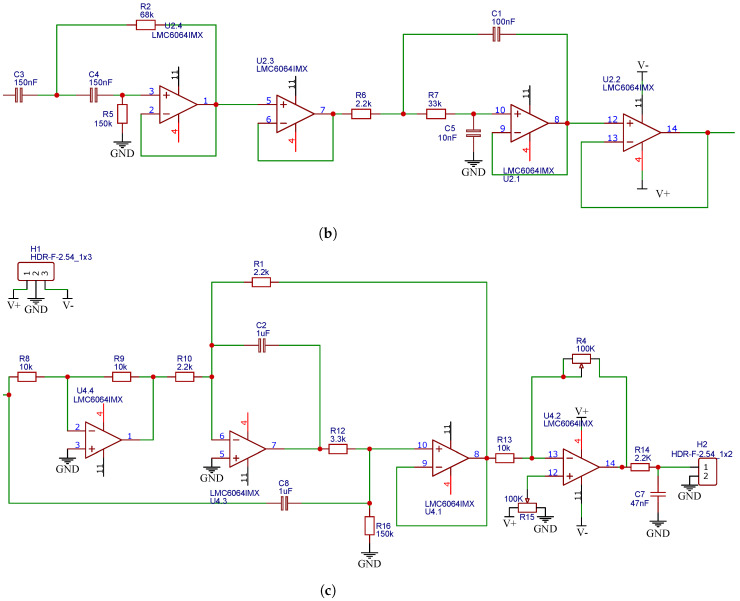
Electrical diagram of the complete EMG acquisition system. (**a**) The instrumentation amplifier is shown with the operational amplifier in integrating configuration, while in (**b**), the low-pass and high-pass filters are found. On the other hand, in (**c**), the notch filter and the offset compensator are exemplified.

After the filtering stage, there is a summing amplifier to compensate for a possible direct current voltage that was not eliminated by the feedback between the output and the reference of the instrumentation amplifier through the op-amp in the integrating configuration. The signals recorded by the acquisition cards were digitized using the National Instruments USB-6002-16-bit acquisition system for later sending to a personal computer through the USB protocol. The final electrical schematic of the EMG system is presented in Figure 1

**Figure 2:**
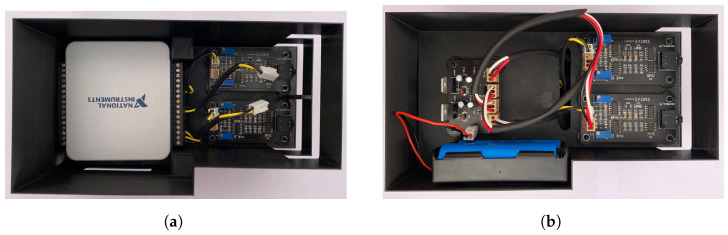
View (**a**) of the first floor of the EMG acquisition system where the two acquisition channels and the power supply system are located, and (**b**) the second floor of the EMG acquisition system where the two remaining channels and the acquisition system are located.

For the power system, a TC7660SCPA switching voltage regulator has been used to power the system with two 3.4 V Li-ion rechargeable batteries in series. The TC7660SCPA regulator, in conjunction with a 7805 voltage regulator, is responsible for generating the negative part (−5 V) from a dual source, while a 7805 voltage regulator generates the positive output (+5 V). Finally, the surface mount acquisition system PCB was completed in EasyEDA^®^, using a 1206 packaging for the passive elements and SOIC packaging for the op-amp and instrumentation amplifier. The two parts of the EMG system are represented in Figure 2.

**Figure 3:**
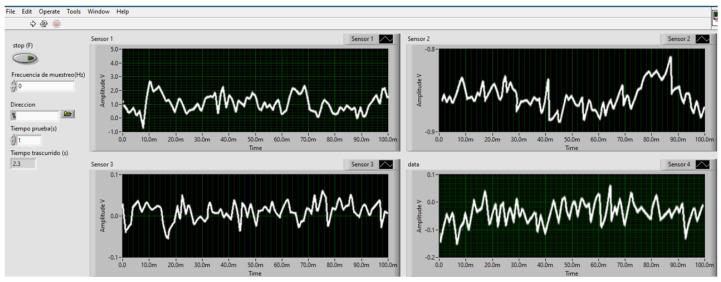
Window with the 4 graphs for each channel in the user interface.

On the other hand, a user interface was designed to control and monitor the acquisition system. It was carried out in NI LabVIEW 2021^®^. This interface consists of 4 graphs in real time for the visualization of the four channels connected to the DAQ USB-6002, a space for selecting the test frequency, a section for the name and address of saving the file, and finally, a section for the duration of the test. An executable (.exe) was created whose only requirement is that the computer where it is executed has LabVIEW Runtime. In Figure 3, the user interface is illustrated.

### 3.2. EMG Data

Two databases were used: the first consists of 9 subjects, five men and four women between 21 and 30 years of age, without musculoskeletal and nervous system diseases and amputations or obesity problems. The database comprises seven movements of the right leg (toe-off, heel-off, toe right, toe left, heel strike without toe-off, support of the foot without lifting the heel, and the state of rest). For the experimentation, bipolar electrodes formed four channels with the reference in the knee. The sampling frequency was 1 kHz. The test subject was asked to perform the above movements for 6 s with 1 s relaxation for experimentation. Each movement was repeated 20 times.

Subsequently, the second database consisted of the muscle signals of 9 people between 23 and 27 years of age, five men and four women, without diseases of the musculoskeletal system and nervous system, without amputations or obesity problems. The database comprises five arm and hand movements (arm flexion at the elbow joint, arm extension at the elbow joint, finger flexion, finger extension, and resting state). For the experiment, four bipolar channels and a reference electrode placed in the dorsal area of the wrist were used. The test subject was asked to perform the above movements for 6 s with an initial 2 s of relaxation. Each movement was repeated 20 times using a sampling frequency of 1.5 kHz. Table 2 presents the main characteristics of the databases used.

### 3.3. Feature Extraction

It is necessary to characterize the signals for EMG classification since the values that make up the signal individually have no practical importance for its classification [27]. Therefore, a feature extraction stage is necessary to find helpful information to classify before features from the signal are extracted. The first stage was to obtain segments of the available EMG signals (windows). A window length of 250 ms with an overlap of 190 ms is used. The features in the time domain are based on the statistical method. The main advantage of temporal features is that it is not necessary to change the domain to compute them, which translates into low complexity and fast computation speed. Because of this, TD features are widely used to classify EMG signals [28,29]. TD characteristics were used, which are illustrated in Table 3. Only characteristics in the time domain were used, since they can be calculated using the acquired EMG time series without applying any intermediate transformation, which causes a reduction in processing time. The features in this domain are widely applied because their performance during classification presents a low amount of noise, and their processing time is less than that shown by the features in the frequency domain [2].

The first stage consisted of preprocessing the acquired signals, where the signals were digitally filtered in a bandwidth of 10 to 500 Hz using a digital fourth-order Butterworth filter. After that, the 26 predictors described before were calculated. The features were extracted considering the complete signals and for each of the windows obtained from the original signal. Windows have a duration of 250 ms with 190 ms of overlap.

Once the characteristics were extracted, a matrix arrangement was made of them, resulting in two matrices for each database. The first one corresponds to the characteristics of the unsegmented signals, resulting in a matrix where the rows correspond to the 20 tests × 8 people × the different movements (seven movements for the database of the right leg and five movements for the right arm database) and the columns to the 26 predictors × the 4 channels. The second matrix corresponds to the segmented signals. This time, the rows correspond to the number of windows × the 20 repetitions × 8 people × for the different movements (seven movements for the database of the right leg and five movements for the right arm database) and the columns correspond to the 26 predictors × the 4 channels.

### 3.4. Design and Integration of the Genetic Algorithm and SVM

Through the training process, SVM finds a hyperplane capable of distinguishing various categories. The main problem in the definition of the SVM model is to determine the function of the kernel and the values of its parameters that best suit the problem to be solved [1,30]. The training process is the solution to an optimization problem using quadratic programming. Thus, the solution found is unique and global. Given the input data $\left\{\left(x_{1},x_{2}\right)\ldots \left(x_{n},x_{m}\right)\right\}\in R^{n}\times \left\{1,-1\right\}$ where $x_{i}$ is the input value and $y_{i}$ is the class to which {1 or −1} corresponds, which belongs to a binary output. Note that if these coordinate values cannot be linearly separated, a nonlinear transformation ϕ: $R^{N}\to R^{M}$ is applied. In this new higher dimensional space, the data are linearly separable. Obtaining a hyperplane that separates the classes, Equation (1) represents the previously described [2,30]:

(1) ω·ϕ+b=0

where $\omega \in R^{M}$ and b∈R.

Since data in most applications are not linearly separable, no hyperplane perfectly discriminates between classes. Therefore, the goal is to find the hyperplane with the lowest error, which has both a maximum separation value and a closed error $\zeta =(\zeta_{1}\ldots \zeta_{m})$, resulting in [2,30]:

(2) $$min\frac{1}{2}{\left||\omega |\right|}^{2}+C\sum_{i=1}^{m}\zeta_{i}$$

where *C* is a regularization parameter for the weighting of the penalty term in the objective function and $\zeta_{i}$ is the slack variable for each training instance $x_{i}$.

If $x_{i}$ is correctly sorted, then i=0. If $x_{i}$ falls within the margin or is misclassified, then *i* is set to the distance of $x_{i}$ in the separating hyperplane. The constraints of the optimization problem become [2,30]:

(3) $$y_{i}\left(\omega \cdot \phi \left(x_{i}\right)+b\right)\geq 1-\zeta_{i},i=1\ldots m.$$

When the data are too close, it becomes difficult to establish the separation. This is when a *K* kernel function must be used to separate them:

(4) $$F\left(\alpha \right)=\sum_{i=1}^{m}\alpha_{i}-\frac{1}{2}\sum_{j,k=1}^{m}\alpha_{j}\alpha_{k}y_{j}y_{k}K\left(x_{j},x_{k}\right)$$

where $K(x_{j},x_{k})$ is the kernel function, which can be an RBF, a Gaussian, a polynomial, etc.

On the other hand, genetic algorithms have been widely used to solve optimization problems. These are based on natural selection, with the chromosome being a possible feature vector of genes representing each element. An initial population with several chromosomes is randomly formed, and new populations are produced through the iterative use of genetic operators on individuals in the initial population. Among the main genetic operators are selection, crossing, mutation, and the calculation of the fitness function [31].

As mentioned above, to start the optimization process by genetic algorithms, it is necessary to establish an initial population *P* with *i* number of elements, named chromosomes, which in turn have *j* number of genes, and population *P* must be formed randomly. Once the population *P* is established, the aptitude of each chromosome is calculated, and the vector of characteristics achieves the classification percentage.

Once the fitness level has been calculated to optimize the task, two chromosomes are selected based on their fitness. The crossover operator is applied to these elements, which produces offspring, in other words, a new feature vector $C_{1}$. Once the crossing is over, the mutation operator is applied to the offspring, which establishes a mutation probability to generate $C_{2}$, the new offspring $C_{2}$ is placed in a new population, and the selection, crossing, and mutation process are maintained in an iterative process over the population *P* until a new population $P_{2}$ [31] is generated.

The algorithm is iterative, so it stops until the number of repetitions is reached or until the desired accuracy value is reached. Ultimately, the best overall chromosome is selected as the subset of the best features [32].

Genetic algorithms use a variety of operators during the search process: crossover, mutation, and selection schemes.

At the beginning of the feature optimization stage, an ANOVA statistical analysis was implemented. Through the box plots, it was verified that the statistical distribution of the data of a complete signal, which has a duration of 5 s, was similar to the distribution of data from the windows, which have a duration of 250 ms, to use the complete signals to select the features that were then used to classify the windows. When all the windows resulting from the segmentation are used, the selection process takes too long, approximately 3 h per iteration of the genetic algorithm. Additionally, ANOVA results were used to remove features that did not present a relevant statistical difference for the right-leg database. On the other hand, the total characteristics for elimination by genetic algorithms for the base of the right arm were entered.

**Figure 4:**
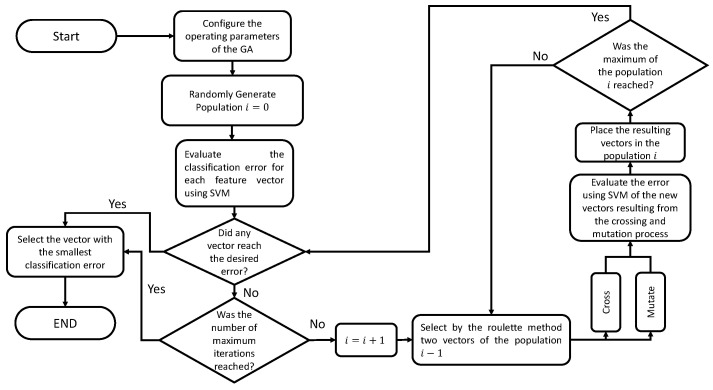
Flow chart for the integration of genetic algorithms and SVM for feature selection algorithm.

The genetic algorithm was applied to minimize the average classification error using the average error calculated through cross-validation, dividing the total data into ten subsets as a cost function to be minimized, as shown in Figure 4. The implemented classifier was multi-class SVM: one against all using the Gaussian and linear kernel functions. It is worth mentioning that 70% of the complete signals were used to select parameters and train the SVM. At the same time, the remaining 30% was reserved for validating the preliminary efficiency percentages of the algorithm.

In this work, simple GA is used to optimize the classification percentage given by SVM. The operators used for the genetic algorithm were:

- Selection operator: roulette wheel;
- Crossover operator: two points;
- Mutation operator: uniform mutation.

### 3.5. Binary Particle Swarm Optimization

A PSO algorithm works with a population (called a cloud or swarm) of candidate solutions (called particles). These particles will move along the search space according to a simple mathematical rule. The movement of each particle depends on its best position obtained and on the best global position found in the entire search space. As new and better positions are discovered, they go on to orient the movements of the particles. The process is repeated with the non-guaranteed objective of finding a sufficiently satisfactory solution [32]. At each iteration, the velocity of each particle is updated as shown in Equation (5).

(5) $$v_{id}^{t+1}=wv_{id}^{t}+c_{1}r_{1}\left(pbest_{id}^{t}-X_{id}^{t}\right)+c_{2}r_{2}\left(gbest_{d}^{t}-X_{id}^{t}\right)$$

where *v* is the speed, *w* is the inertial weight, $c_{1}$ represents cognitive learning and $c_{2}$ social learning, $r_{1}$ and $r_{2}$ are independent random vectors in [0,1], *X* is the solution, and pbest is the personal best solution. At the same time, gbest is the best global solution present in the entire set of solutions, *i* is the order, *d* is the dimension of the search space, and *t* is the number of iterations. The sigmoid function is used to convert the velocity to the probability value, as shown in Equation (6).

(6) $$S\left(v_{id}^{t+1}\right)=\frac{1}{1+e^{-v_{id}^{t+1}}}$$

The new position of a particle is updated based on the probability value as follows:

(7) $$X_{id}^{t+1}=\left\{\begin{array}{ccc} 1 & if & S\left(v_{id}^{t+1}\right)>r_{3}\\ 0 & if & otherwise \end{array}\right.$$

where $r_{3}$ is a random number distributed between 0 and 1. PSO is widely used in binary optimization problems. However, PSO has various issues, such as premature and slow convergence rates. In addition, the adjustment of the inertia weight is often complicated to perform.

### 3.6. Classifier Performance Analysis

On multiple occasions, large data sets are not available to the researcher. On the other hand, small databases cannot be partitioned efficiently into a relevant training set and a reliable test set. This is because the statistics detected depend on how the training and test sets are formed. One way to solve these difficulties is through cross-validation (*k*-fold), since it allows the results to be significantly robust.

The method divides the database into *k* non-overlapping subsets that will be used as training and validation sets. Subsequently, the classification algorithm is trained *k* times. In each iteration, one subset is used as the new test set, while the remaining subsets make up the training set. The false positives and negatives for each of the iterations performed by the system are used to calculate: the precision Equation (8), the specificity Equation (9), the sensitivity Equation (10), and the efficiency Equation (11).

(8) $$Accuracy=\frac{TP+TN}{TP+TN+FP+FN}$$

(9) $$Sensitivity=\frac{TP}{TP+FN}$$

(10) $$Specificity=\frac{TN}{TN+FP}$$

(11) $$Efficiency=\frac{Accuracy+Sensitivity+Specificity}{3}$$

*TN* is the true negative case, *FN* is the false negative case, *TP* is the true positive case, and *FP* is the false positive case. The average of the *k*-trained models is calculated to obtain the total efficiency [33].

Finally, a final SVM was trained with the reduced feature space of the database in [25]. The proposed efficiency of algorithm levels was checked using 30% of the windows for the training and 70% for the final validation. Subsequently, the algorithm was applied to its own database. Once the feature vector was selected, an SVM was trained with 25% of the windows, and 75% was used to validate the performance.

The final performance of SVM was evaluated by calculating the efficiency, accuracy, sensitivity, and specificity derived from the confusion matrix.

### 3.7. Sensitivity Analysis

One way to determine the impact each feature has on the prediction of the model is through sensitivity analysis. The sensitivity is calculated by changing the value of a predictor. In this case, it is removed from the classification process. In contrast, all other features remain constant, and the model error is evaluated. If removing the feature drastically alters the output of the model, it means that it has a significant impact on the prediction [34]. This procedure is realized once the features have been selected to evaluate the importance of the selected predictors through PSO and GA.

Given a test set $X_{1}$, the sensitivity of feature *i* is calculated by creating a new set $X_{2}$ where predictor *i* is removed. Subsequently, the evaluation of $X_{1}$ is carried out, giving as a result, the accuracy $Y_{1}$. The third step consists of calculating the accuracy of a new vector $X_{2}$ and obtaining $Y_{2}$. The sensitivity for feature *i* will then be $Y_{2}-Y_{1}$. Another way to see the impact of the feature on the accuracy is to calculate the percentage change of the new accuracy concerning the accuracy given by the vector $X_{1}$, which is calculated as:

(12) $$Percentage\;change=\frac{Y_{2}-Y_{1}}{Y_{1}}\times 100$$

## 4. Results

This section presents and analyzes the results obtained from the different stages of the proposed selection and classification algorithm methodology.

### 4.1. Filtering

**Figure 5:**
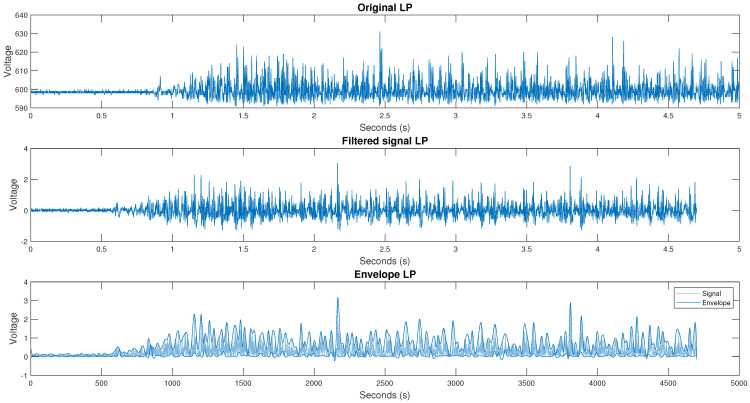
Original, filtered, and surround signal from channel 1, person 1, and test 1 for the toe-off movement.

**Figure 6:**
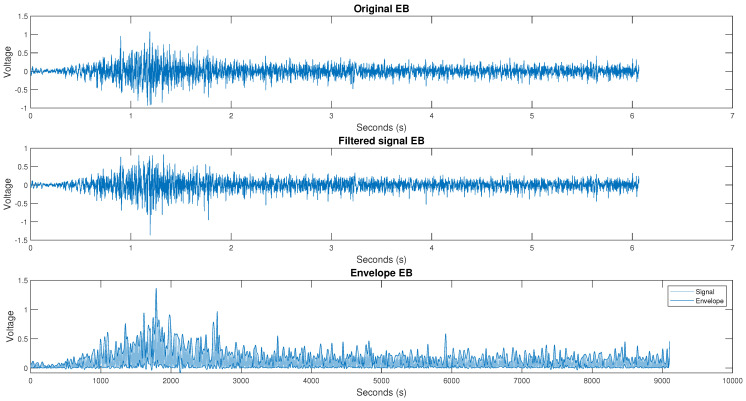
The original, filtered, surround signal from channel 2, person 1, and test 1 for arm extension movement.

This section describes the filtering and segmentation process of the databases used, the first being from the lower right extremity and the second the database from the upper right extremity. As mentioned above, the first stage after acquiring the signals is to filter them. The filtering was carried out using a fourth-order Butterworth bandpass filter with cutoff frequencies from 10 to 500 Hz. An example of the results of the filtered signals for the base data from the right leg is illustrated in Figure 5. The filtering procedure was repeated for the second database using the fourth-order Butterworth filter with cutoff frequencies of 10 to 500 Hz. Figure 6 shows the result of the right arm filtered signals of the database.

### 4.2. Segmentation

Once the filtering part was finished, the signals were divided into windows of 250 ms with an overlap of 190 ms, 64 windows per signal were obtained for the database corresponding to the right leg. On the other hand, 147 windows were calculated for the right-arm database.

Overlapping windows were used due to it allows information to be acquired, while the classification algorithm works, facilitating real-time application. Furthermore, the overlapping windows increase the frequency of classification decisions, since the analysis windows require fewer data to complete the total time, which is 250 ms.

**Figure 7:**
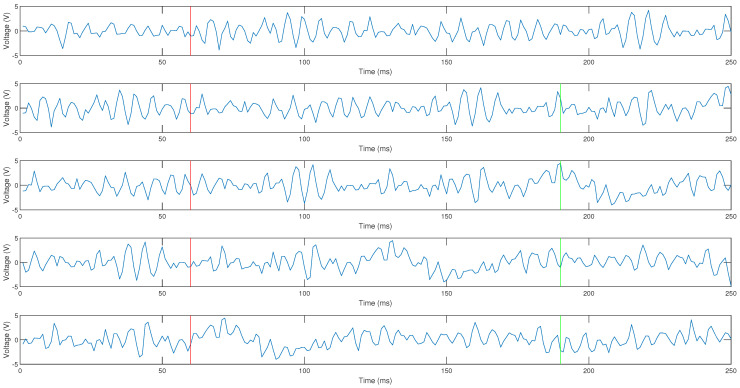
From top to bottom, windows 40, 41, 42, 43, and 44 of the signal of channel 1 of the person 1 of the repetition 1 of the EMG signals of the right leg are represented.

The following reasoning was applied to form the windows: first, an initial window of 250 ms is formed. The time interval data from 60 to 250 ms is taken to form the second window. The interval is shown in Figure 7 between the red line and the end of window one. This time, the interval forms the first 190 ms of window two, which is marked by the green line. From this line, new data correspond to 60 ms. The data are used in the interval marked by the red line of window two until the end of the signal from to form window three, these being the first 190 ms of window three (green line). The remaining 60 ms is filled with new data. This process is repeated until the total number of windows required is formed.

### 4.3. Feature Extraction

Once the segmentation of the windows was completed, the characteristics in the time described in the theoretical foundation section were extracted.

ANOVA was applied to see the data distribution since, to select the classification parameters by genetic algorithms, it is necessary to train the SVM multiple times to measure the average error percentage of the feature spaces given by GA, so using the segmented signals would slow down the process due to the large amount of data involved. However, since the distribution of data per window is similar to the distribution corresponding to the unsegmented signals due to the application of overlapping windows, it is possible to use the data without the segmentation process for selecting classification parameters.

**Figure 8:**
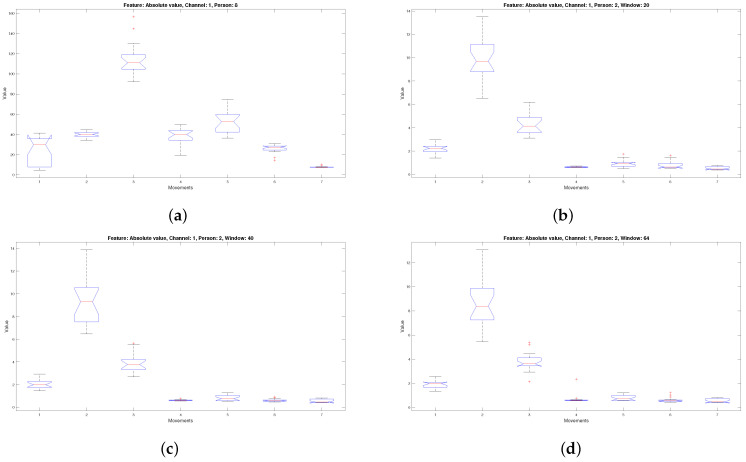
Box plot of the absolute value characteristic corresponding to the seven movements of channel one of person two (**a**) considering the unsegmented signal, (**b**) considering window 20, (**c**) considering window 40, and (**d**) considering window 64.

Figure 8a illustrates the distribution corresponding to the characteristic of the mean of the absolute value for person two channel one of the databases of the lower right extremity; when compared with Figure 8b–d, which consider the absolute value for person two channel one windows 20, 40 and 64, it is seen that they have a similar data distribution.

**Figure 9:**
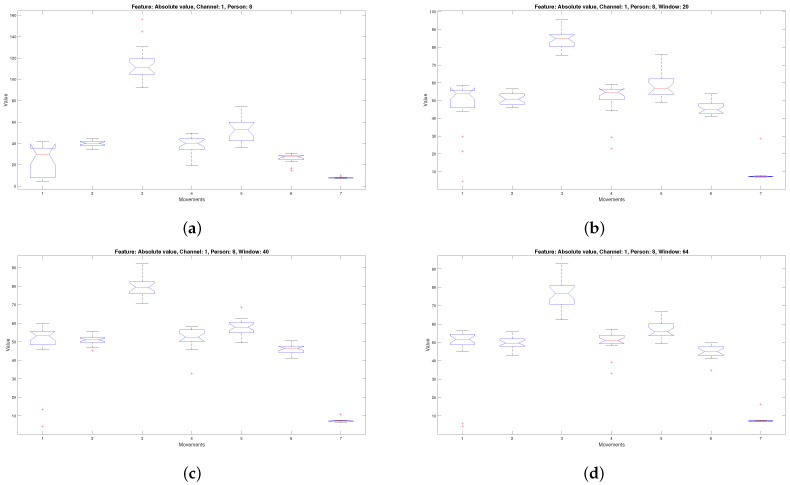
Box plot of absolute value characteristic corresponding to the seven movements of channel one of person eight (**a**) considering the unsegmented signal, (**b**) considering window 20, (**c**) considering window 40, (**d**) considering window 64.

The same happens with Figure 9a–d. A comparison process of distributing the data corresponding to windows 20, 40, and 64 against the data coming from the complete signals was carried out for the 26 predictors from each channel corresponding to the eight people. Windows were used: 20, 40, and 64, because they correspond to the initial, intermediate, and final windows, which allows us to make a comparison that encompasses different times within the acquired signal.

Once the process of comparing the distribution of the data of each one of the predictors corresponding to the windows against the complete signals was completed, the value *p* was used to verify if there are statistically significant differences between the means of the classes if the value *p* of the ANOVA test was more significant than the alpha significance value (α=0.05), so the means of the groups were equal and were eliminated.

After the analysis, 24 characteristics were eliminated, corresponding to six predictors: average amplitude value, absolute standard deviation difference, fractals, entropy, Willson amplitude, and myopulse percentage rate, resulting in two arrays. The first one corresponds to the characteristics of the unsegmented signals, having a dimension of 1120×80, with the rows corresponding to 8 people × 7 movements × 20 repetitions and the columns four channels × 24 features. The second matrix corresponds to the segmented signals. The matrix has dimensions of 71,680×80. The rows are made up of 8 people × seven movements × 20 repetitions × 64 window. On the other hand, the columns did not undergo modification.

Although ANOVA is a method used for the selection of features [35,36,37], it has certain disadvantages, since the ANOVA analysis only allows us to observe if there is a difference between the classes but does not refer to the contribution to the classification; a characteristic that presents a statistical difference between the different classes could not present an individual contribution to the classification or in the same way could not represent a contribution together with the other sort parameters [38]. Furthermore, it only increases the complexity of the system without giving a significant advantage against other feature spaces. In this sense, genetic algorithms make it possible to validate the overall contribution of the different classification parameters since, based on a conventional and statistical analysis method, the characteristics that hinder the classification process have a greater chance of being eliminated. In contrast, those that favor a higher classification are chosen within the final parameter space.

On the other hand, ANOVA was not applied to the second database for feature removal, since the power of genetic algorithms to select features without prior review and remove features needed to be analyzed.

Finally, two matrices were obtained for the second database. The first one corresponds to the characteristics of the unsegmented signals, having a dimension of 800×104, with the rows corresponding to 8 people × 5 movements × 20 repetitions and the columns 4 channels × 26 predictors. The second matrix corresponds to the segmented signals. The matrix has dimensions of 11,7600×104. The rows are made up of 8 people × 5 movements × 20 repetitions × 147 windows. On the other hand, the columns did not undergo modification.

### 4.4. Feature Selection

The feature selection subsection comprises two stages. The first corresponding to the analysis based on data from the right leg. In contrast, stage two consists of applying the algorithm developed based on data from the right upper extremity.

#### 4.4.1. Feature Selection for the Right Lower Extremity Database

The following experimentation parameters were defined, which are indicated in Table 4 and Table 5, where the kernel was modified alongside the number of iterations, the percentage of mutation, and the number of initial vectors to know the contribution or importance of each of the mentioned variables. Hence, in the first phase, the database corresponding to the right leg was used.

**Figure 10:**
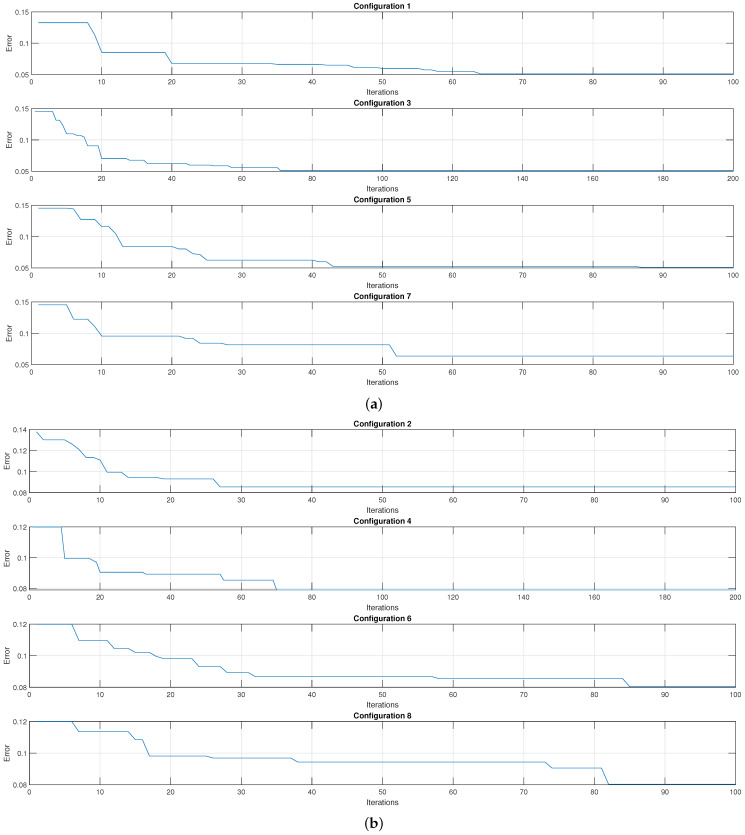
Plot of the classification percentage as a function of the number of iterations for (**a**) the Gaussian kernel and (**b**) the linear kernel.

Figure 10 presents the graphs that relate the result of the cost function of the genetic algorithm $\left(\frac{\%Classification}{100}\right)$ and the iteration number in which it was obtained for each of the configurations. In the beginning, there is an average classification error given by cross-validation close to 14% using the Gaussian kernel. When reaching the final iteration, the cross-validation gives an average error close to 5%. On the other hand, when using the linear kernel, there is an initial average classification error of close to 12% and an absolute average error of 8%.

**Figure 11:**
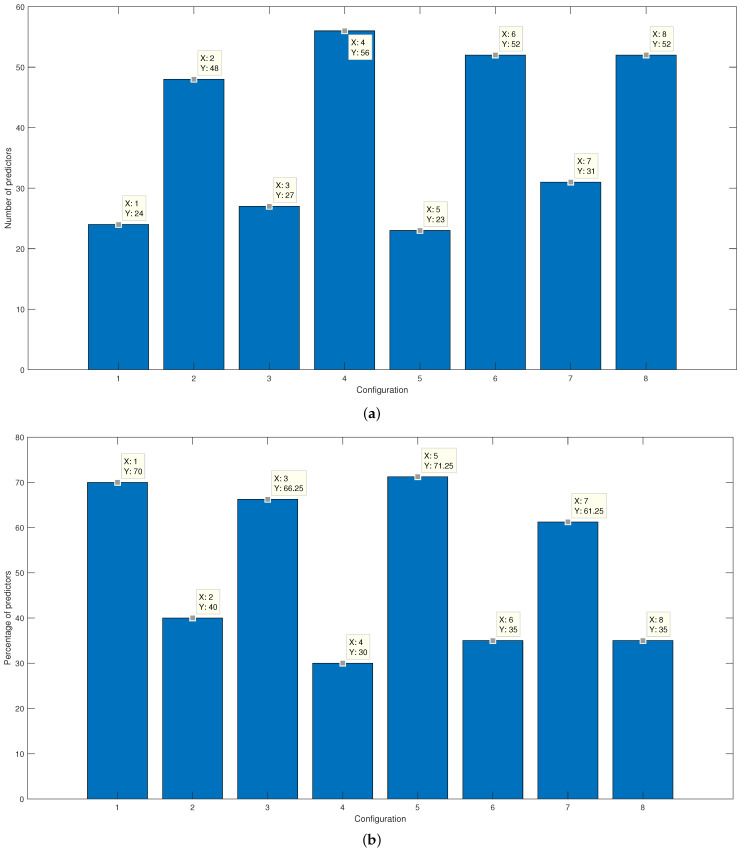
Results of the average error percentage for the training and validation of the final SVM with (**a**) Gaussian kernel and with a (**b**) Linear kernel.

Figure 11a indicated the final number of features in each of the configurations. On the other hand, in Figure 11b, the percentage corresponding to the reduction of characteristics due to the selection process is seen. It is essential to consider that using the Gaussian kernel makes it possible to reach an absolute mean error similar to that of the linear kernel. However, the number of features needed to reach a similar classification percentage is higher when using the linear kernel.

**Figure 12:**
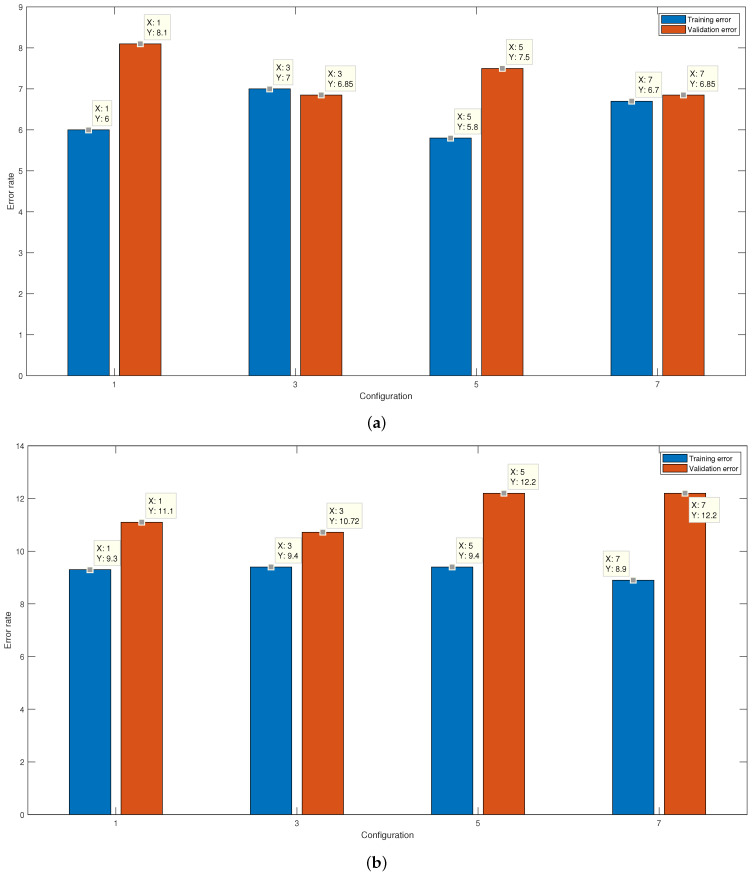
Results of the average error percentage for the training and validation of the final SVM with (**a**) Gaussian kernel and with a (**b**) Linear kernel.

Once the feature space that provides the lowest average classification error was calculated using 70% of the signals, the remaining 30% of the data was used for validation. In Figure 12a, the results of the average error of the training against the average error in the validation are shown for each of the configurations in which the Gaussian kernel was used. On the other hand, Figure 12b unfolds the results for the linear kernel.

When comparing the different configurations, the Gaussian kernel similarly classifies the data to the linear kernel but using a smaller number of features. Therefore, it was decided to use the Gaussian kernel to classify the windows, taking the features of configuration 3, which is represented in Table 6, since it is the one that presented a smaller difference between the average meddling error and the average validation error for the unsegmented signals. Table 7 highlights the results for 70% of the windows in the SVM training stage. On the other hand, Table 8 shows the operating parameters for the 30% of the remaining windows in the validation phase.

#### 4.4.2. Selection of Features for the Database of the Upper Right Extremity Using GA

Once the analysis of the proposed algorithm was completed using the right-leg database, it was applied to the right-hand database to check its repeatability. Considering that for all configurations, a similar final average error was reached due to the contribution of each parameter, it was decided to use the configuration illustrated in Table 9 where the number of genes is given by 26 predictors × 4 channels.

**Figure 13:**
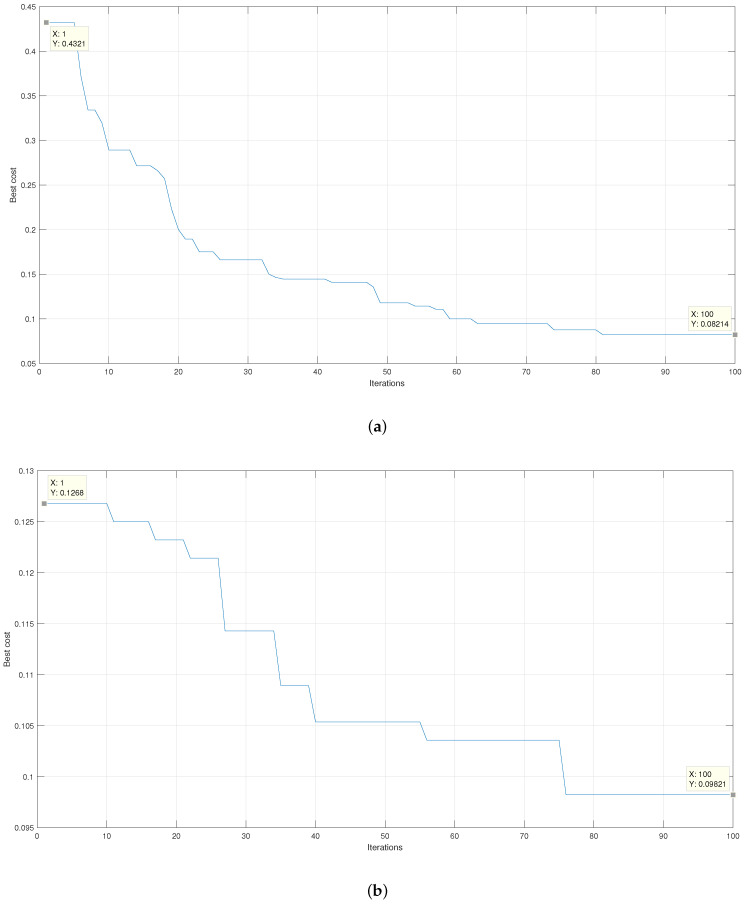
Results of the average error percentage for the training and validation of the final SVM with (**a**) Gaussian kernel and with (**b**) linear kernel.

Figure 13 presents the results of the average error reduction obtained during the feature selection process. It is appreciated that for the linear kernel in Figure 13b, there is an initial average error of 12.68%, which is less than the initial average error presented by the algorithm when using the Gaussian kernel represented in Figure 13a, which is 43.21%. However, when the Gaussian kernel is applied, an absolute average error of 8.21% is obtained, while when using the linear kernel, an absolute average error of 9.82% is obtained. The result obtained is using 70% of the unsegmented signals. When both results are compared, it is observed that when the Gaussian kernel is applied, there is a faster and more significant decrease in the magnitude of the initial average error.

**Figure 14:**
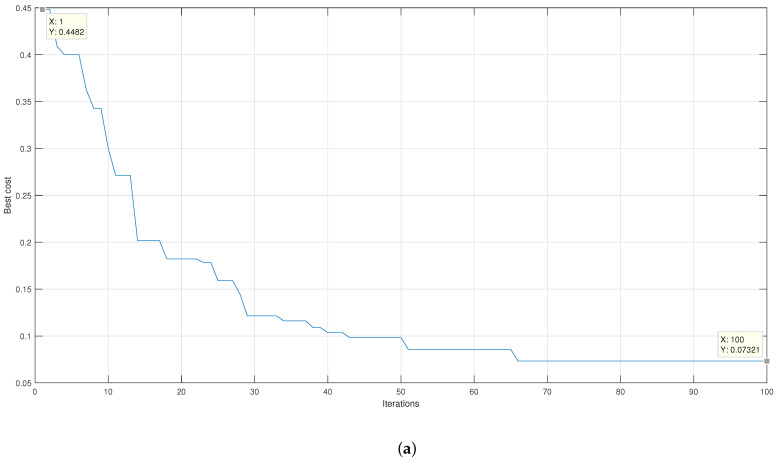

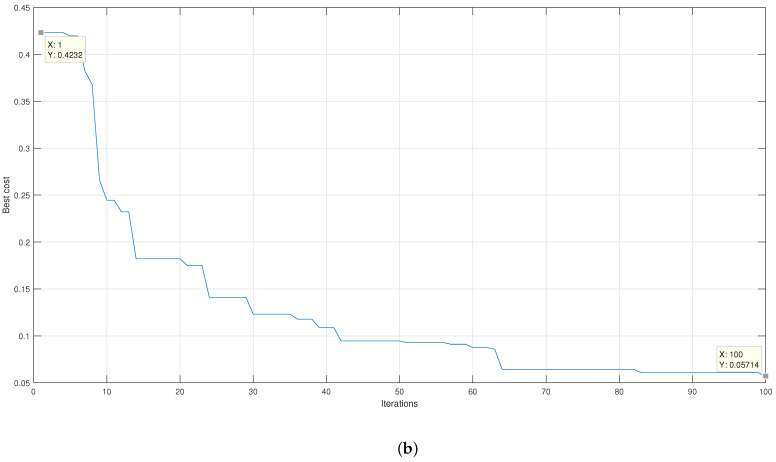
Graph of the classification percentage as a function of the number of iterations using the Gaussian kernel for (**a**) repetition 2 and (**b**) repetition 3.

Table 10 presents that to achieve a similar average classification error, the linear kernel SVM uses significantly more functions than the Gaussian kernel SVM. Due to the low number of features needed to classify, the Gaussian kernel was used. The selection was repeated twice with the same genetic algorithm settings and using only the Gaussian kernel to demonstrate repeatability. The results of the feature selection are illustrated in Figure 14. As shown in Figure 13a,b, the initial average error decreases rapidly. It has a similar initial magnitude close to 43%, while the final average error ranges from 5% to 7%. The number of characteristics for replica 2 was 14, while in replica 3, 12 characteristics were obtained. Table 11 shows the characteristics of the best subset selected by GA.

The parameters given by repetition one were taken to classify the windows to finish the feature selection stage because said repetition presented the least number of features to reach a final average error similar to the other two repetitions. For the classification of the windows of the upper right extremity, 25% of them were used in the SVM training stage. The results are presented in Table 12. On the other hand, Table 13 presents the operating parameters for 75% of the remaining windows in the validation phase.

The classification of 14,700 windows from a person who was not included in the selection and training phase of the SVM was carried out. The sensitivity, specificity, accuracy, and efficiency results are illustrated in Table 14. It is observed that the efficiency for the resting state and the extension of the fingers decreased the most.

### 4.5. Selection of Features for the Database of the Upper Right Extremity Using PSO

In order to contrast the application of genetic algorithms in selecting characteristics for EMG signals, a comparison is made with another popular method known as PSO. For this comparison, only the database corresponding to the right upper extremity was used. The implemented PSO algorithm is based on the [39] algorithm. Table 15 and Table 16 show the values of the variables used for the PSO configuration in the characteristics selection process, in which the complete signals were used.

**Figure 15:**
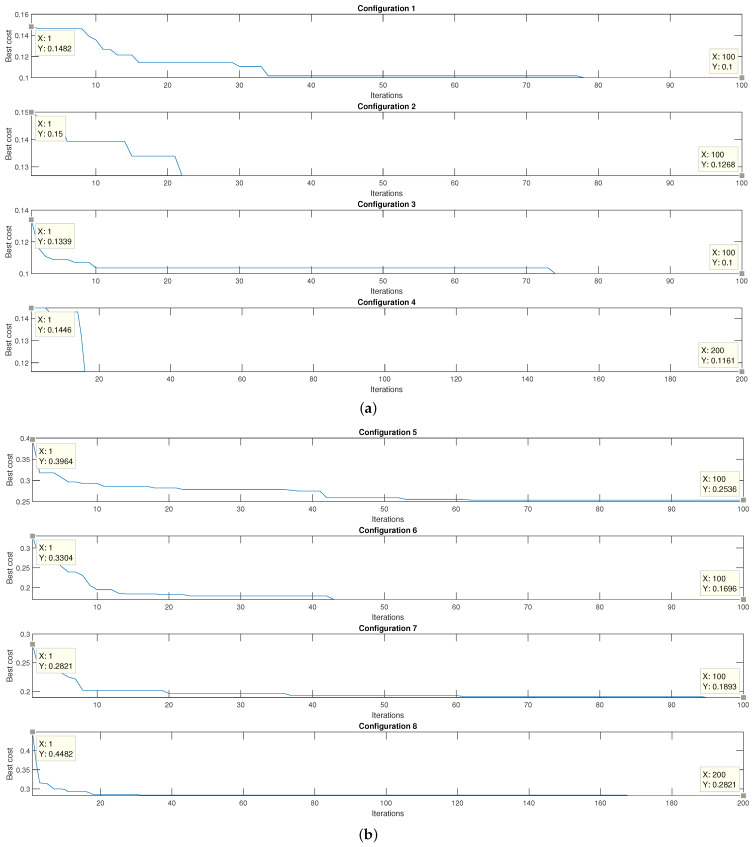
Plot of the classification percentage as a function of the number of iterations for (**a**) the linear kernel and (**b**) the Gaussian kernel.

The error values achieved for the various PSO configurations are shown in Figure 15. Figure 15a shows that the average error value reached is 11%. On the other hand, as illustrated in Figure 15b, the average error value reached for the four configurations was 22.3%.

**Figure 16:**
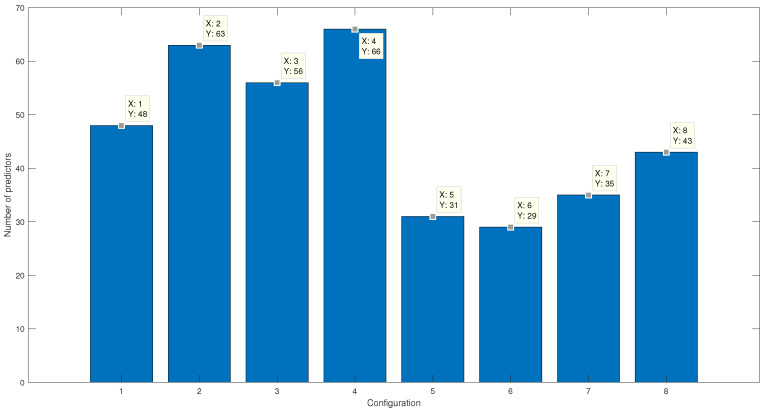
The number of features given by PSO using the SVM error as a fitness function for the various configurations.

Figure 16 shows the number of features selected for the various configurations. A more significant number of features is used for the linear kernel, while for the Gaussian kernel, approximately half of the features are required to carry out the predictions.

Once the selection stage was completed, the result obtained from one of the configurations was used to classify the windows. Specifically, the characteristics given by configuration one were used because it presented the slightest error given the least number of selected parameters for the linear kernel. Table 17 presents the characteristics selected by PSO as the best subset for the classification of movements. For the classification of the windows of the upper right extremity, 25% of them were used in the SVM training stage. The results are presented in Table 18. On the other hand, Table 19 presents the operating parameters for 75% of the remaining windows in the validation phase.

#### Sensitivity Analysis

**Figure 17:**
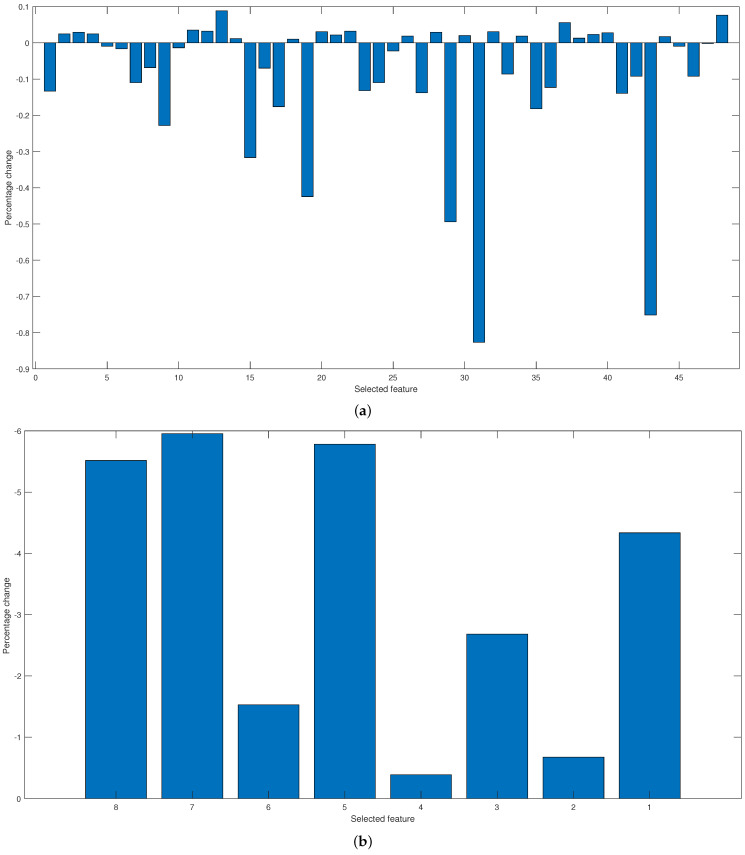
Plot of the classification percentage as a function of the number of iterations for (**a**) the linear kernel and (**b**) the Gaussian kernel.

Finally, the sensitivity analysis was applied to evaluate the importance of the characteristics selected by PSO and GA. Figure 17a shows the percentage change of the parameters selected by PSO, while Figure 17b illustrates the percentage change of the characteristics given by GA.

## 5. Discussion

As shown in the last section, genetic algorithms are a powerful tool for feature selection. For this work, single-objective genetic algorithms were used, with the fitness function as the minimization of the average error given by SVM. For each iteration in the feature selection process, a prediction model based on SVM is calculated for each chromosome in the population. Through cross-validation with *k* = 10, the average error from each *k*-validation process is calculated. The feature array or chromosome with the slightest error is selected at the end of the selection process. However, the optimization of sensitivity, precision, and specificity is a consequence of reducing the error in the classification. In other words, the sensitivity, precision, and specificity are maximized by minimizing the error. Since the direct optimization of the previous variables is not sought, some of them may have a significantly lower percentage than another. Genetic algorithms, in conjunction with SVM as a fitness function, proved to have the ability to reduce a feature space, improving the algorithm’s classification capacity. Similarly, due to the combinatorial nature of genetic algorithms, there are non-relevant specific features that do not differ from the classification but do not provide the necessary information, which could be eliminated by analyzing the results of various tests and the experience of the researcher.

Furthermore, when increasing the population size for SVM with the linear kernel, there is a minimum in a greater number of iterations. On the other hand, the opposite happens for the Gaussian kernel, which may indicate that beyond the initial size, the speed at which it reaches a minimum depends on the random configuration of the available chromosomes in the population since, for both cases, SVM with linear kernel and SVM with Gaussian kernel, the final error is comparable to the selection processes with 100 chromosomes in the population. Similarly, the number of selected traits was not affected by population size.

SVM was selected as a classification algorithm because it is a well-established and widely used technique in machine learning problems focused on processing and classifying EMG signals due to its simplicity and robustness. Similarly, it was taken into account that the availability and quality of EMG signals can vary from one volunteer to another, making it difficult to obtain many training samples. In these situations, SVM is suitable for solving learning tasks where the number of attributes is large relative to the number of training examples.

Another critical point to highlight is that depending on the kernel used, different amounts of information are needed to classify so that an erroneous kernel selection could lead to a more complex classifier. Under this dilemma, the presented algorithm indirectly helps to select a kernel under the criterion of choosing the kernel that gives us the lowest average error with the fewest number of parameters.

Although the Gaussian kernel gave the least number of features, the difference between the results of the two databases indicates that the more complex the movement and the more classes, the more information is needed. There were seven classes for the leg database, and the movements were compound. More than two muscles were used to carry out the movement. On the other hand, for the database of the right arm, there were only five movements, and the movements were simple. That is, each one of the registered muscles was responsible for a single movement. It is important to note that since the statistical distribution of the windows and the complete signal are similar, it is possible to use the information without segmentation to select the parameters to later validate with the segmented data, which saves time.

Additionally, when comparing the results given by PSO and GA shown in Figure 13 and Figure 15, respectively, one of the main differences is that the errors reached by GA are lower than those calculated by the characteristics proposed by PSO. On the other hand, the speed of convergence of PSO is faster. However, the number of features selected by GA is 83.33% less than that of PSO. Another significant difference was that the best result was obtained for GA using a Gaussian kernel, while for PSO, the best result was obtained with a linear kernel. Despite that, the Gaussian kernel for both selection methods uses minor information to achieve the classification.

In addition, when performing the analysis of Table 12, Table 13, Table 18 and Table 19, it is observed that GA presents an average sensitivity of 84.38%, an average specificity of 96.1%, an average accuracy of 93.76%, and an average efficiency of 91.42% for the learning stage. In comparison, for the validation stage, it presents 85%, 96.24%, 94%, and 91.7%, respectively. On the other hand, PSO presents an average sensitivity of 76.43%, an average specificity of 94.1%, an average accuracy of 92.4%, and an average efficiency of 87.07% for the discovery stage. The validation stage presents 76.71%, 94.17%, 90.68%, and 87.18%, respectively. Both in the training and validation sections, the parameters selected by PSO offer lower classification percentages than those given by GA.

On the other hand, the sensitivity analysis shows that the eight parameters selected by GA are representative of carrying out the classification since by eliminating any of them in the classification of windows, the percentage of accuracy decreases, with characteristics 4 and 3 showing less decrease in the classification when eliminated. On the contrary, for the characteristics given by PSO, the percentage of change for all the characteristics is less than 1%. That is, when eliminating any of the parameters, the classification is slightly altered.

## 6. Conclusions

The algorithm based on genetic algorithms and SVM achieved an average efficiency percentage of 91% for both databases after the feature selection process. A modular portable EMG signal acquisition device has been designed, developed, and manufactured with surface mount components to reduce the space used. The need for analog filters in the acquisition stage proved to have comparable results to digital filters, which is why they are an option to reduce preprocessing time. The selection of EMG time features overcomes the impact of perturbations on EMG pattern classification to some extent. However, exploring new methodologies for electrode locations is suggested to improve the robustness of EMG pattern recognition further. Genetic algorithms, in combination with SVM as a cost function, have been shown to have the ability to decrease the initial feature space for EMG signals while increasing the percentage in the classification.

Another point to highlight is that both the characteristics given by GA and PSO have problems achieving the classification of the ED movement, which could indicate that the time characteristics representing this class are similar to those of the other movements. Given the above, the efficiency levels will be analyzed in future works by incorporating characteristics in the frequency domain.

Finally, according to the results obtained with GA and PSO for the right-hand database, the characteristics provided by GA exceed those given by PSO not only in the classification levels and efficiencies but also in the number of parameters necessary to carry out the classification. Presenting as the only advantage of the selection by PSO is that the convergence speeds are faster. Table 6 shows the best set of characteristics selected by GA for the right lower extremity. Table 11 and Table 17 show the best characteristics for the right upper extremity given by GA and PSO, respectively. Obtaining the best subset for the right extremity is the one presented in Table 11.

## Figures and Tables

**Table 2:** General characteristics of the implemented databases.

Characteristic	Database 1	Database 2
Body member	Right leg	Right arm
Movements	Lift the toe (AP), lift the heel (AT), move the toe to the right (LP), move the toe to the left (LT), land the heel without lifting the toe (PD), support of the foot without lifting the heel (PI) and the state of rest (RR)	Arm Flexion (FB), Arm Extension (EB), Fist Opening (ED), Fist Closing (FD), and Rest (RR)
Participants	8 (five men and four women)	9 (five men and four women)
Sample rate	1 kHz	1.5 kHz
Signal duration	7 s	8 s
Relaxation time before starting the movement	1 s	2 s
Number of repetitions per movement	20	20
Authors	[25]	Own

**Table 3:** The most common time domain indicators in the classification of EMG signals.

N°	Feature Extracted	Abbr.	N°	Feature Extracted	Abbr.
1	Average amplitude change	AAC	14	Variance	VAR
2	Average amplitude value	AAV	15	Wavelength	WL
3	Absolute standard deviation difference	DASDV	16	Zero crossings	ZC
4	Fractals	FC	17	Log detector	LOG
5	Entropy	SE	18	Mean absolute value	MAV
6	Kurtosis	K	19	Mean absolute value slope	MAVSLP
7	Skewness	SK	20	Modified mean absolute value type 1	MMAV1
8	Mean absolute deviation	MAD	21	Modified mean value type 2	MMAV2
9	Willson amplitude	WAMP	22	RMS value	RMS
10	Absolute value of the third moment	Y3	23	Slope changes	SSC
11	Absolute value of fourth moment	Y4	24	Simple square integral	SSI
12	Absolute value of the fifth moment	Y5	25	Standard deviation	STD
13	Myopulse percentage rate	MYOP	26	Integrated EMG	IEMG

**Table 4:** Initial parameters used for the genetic algorithms for experiments with Gaussian kernel.

Name	Configuration 1	Configuration 3	Configuration 5	Configuration 7
Number of genes	80	80	80	80
Number of parents	100	100	200	100
Mutation percentage	2%	2%	2%	8%
Selection operator	Roulette wheel	Roulette wheel	Roulette wheel	Roulette wheel
Crossover operator	Two-point	Two-point	Two-point	Two-point
Mutation operator	Uniform mutation	Uniform mutation	Uniform mutation	Uniform mutation
Max iterations	100	200	100	100
SVM	Gaussian	Gaussian	Gaussian	Gaussian

**Table 5:** Initial parameters used for the genetic algorithms for experiments with Gaussian kernel.

Name	Configuration 2	Configuration 4	Configuration 6	Configuration 8
Number of genes	80	80	80	80
Number of parents	100	100	200	100
Mutation percentage	2%	2%	2%	8%
Selection operator	Roulette wheel	Roulette wheel	Roulette wheel	Roulette wheel
Crossover operator	Two-point	Two-point	Two-point	Two-point
Mutation operator	Uniform mutation	Uniform mutation	Uniform mutation	Uniform mutation
Max iterations	100	200	100	100
SVM	Linear	Linear	Linear	Linear

**Table 6:** Features selected as the best subset of characteristics for classifying signals from the right lower extremity using configuration 3.

Acronym	Channel
IEMG	1, 2 and 3
LOG	All
MMAV2	1 and 3
STD	1, 2 and 4
ZC	All
MAV	2 and 4
SSC	2
WL	2 and 4
K	2
MAD	2
Y3	3
MMAV1	4
SSI	4
Y4	4

**Table 7:** Classifier performance parameters for the training stage.

	AP	AT	LP	LT	PD	PI	RR
Sensitivity	92.62%	89.12%	94.48%	88.03%	83.74%	83.98%	95.38%
Specificity	98.40%	98.09%	98.63%	98.58%	97.83%	97.77%	98.61%
Accuracy	97.56%	96.84%	98.03%	97.02%	95.85%	95.81%	98.15%
Efficiency	96.19%	94.68%	97.052%	94.54%	92.47%	92.52%	97.38%

**Table 8:** Classifier performance parameters for the validation stage.

	AP	AT	LP	LT	PD	PI	RR
Sensitivity	92.99%	89.53%	94.71%	88.40%	83.84%	83.94%	96.59%
Specificity	98.4%	98.16%	98.49%	98.677%	97.86%	97.92%	98.75%
Accuracy	97.67%	96.91%	97.95%	97.23%	95.84%	95.91%	98.44%
Efficiency	96.37%	94.87%	97.05%	94.77%	92.51%	92.59%	97.93%

**Table 9:** Initial parameters used for genetic algorithms.

Name	Value
Number of genes	104
The initial number of parents	100
Mutation percentage	2%
Selection operator	Roulette wheel
Crossover operator	Two points
Mutation operator	Uniform mutation
Max iterations	100
SVM	Gaussian
SVM	Linear

**Table 10:** Total of features selected by genetic algorithms using the Gaussian and linear kernel.

Kernel	Number of Features
Linear	60
Gaussian	8

**Table 11:** Features selected as the best subset of characteristics for classifying signals from the right upper extremity using the Gaussian kernel.

Acronym	Channel
WL	1
LOG	2
SE	2 and 4
MYOP	2 and 4
MMAV1	3
ZC	3

**Table 12:** Classifier performance parameters for the training stage.

	FB	EB	FD	ED	RR
Sensitivity	93.20%	89.57%	84.47%	72.940%	81.75%
Specificity	97.19%	98.39%	94.03%	95.32%	95.59%
Accuracy	96.38%	96.61%	92.14%	90.81%	92.88%
Efficiency	95.59%	94.86%	90.22%	86.36%	90.07%

**Table 13:** Classifier performance parameters for the validation stage.

	FB	EB	FD	ED	RR
Sensitivity	93.40%	90.47%	85.18%	73.83%	82.13%
Specificity	97.47%	98.43%	94.15%	95.31%	95.86%
Accuracy	96.66%	96.84%	92.35%	91.02%	93.10%
Efficiency	95.84%	95.24%	90.56%	86.72%	90.36%

**Table 14:** Classifier performance parameters for the blind validation stage.

	FB	EB	FD	ED	RR
Sensitivity	85.272%	81.939%	80.238%	63.946%	72.143%
Specificity	95.612%	93.206%	95.927%	83.214%	82.925%
Accuracy	93.544%	90.952%	92.789%	86.361%	70.769%
Efficiency	91.476%	88.699%	89.651%	77.84%	75.279%

**Table 15:** Initial parameters used for the PSO for experiments with linear kernel.

Name	Configuration 1	Configuration 2	Configuration 3	Configuration 4
Number of particles	10	20	30	10
Cognitive factor	2	2	2	2
Social factor	2	2	2	2
Inertia weight	0.9	0.9	0.9	0.9
Max iterations	100	100	100	200
SVM	Linear	Linear	Linear	Linear

**Table 16:** Initial parameters used for the PSO for experiments with Gaussian kernel.

Name	Configuration 5	Configuration 6	Configuration 7	Configuration 8
Number of particles	10	20	30	10
Cognitive factor	2	2	2	2
Social factor	2	2	2	2
Inertia weight	0.9	0.9	0.9	0.9
Max iterations	100	100	100	200
SVM	Gaussian	Gaussian	Gaussian	Gaussian

**Table 17:** Features selected as the best subset of characteristics for classifying signals from the right upper extremity using configuration 1 and PSO.

Acronym	Channel
AAC	3
AAV	4
DASDV	1, 2 and 4
FC	2 and 3
IEMG	1, 2 and 3
K	All
LOG	1, 3 and 4
MAD	3 and 4
MAVSLP	4
MMAV1	4
MMAV2	1, 2 and 3
MYOP	1, 3 and 4
RSM	4
SE	1 and 3
SK	2, 3 and 4
SSI	1 and 4
STD	1, 3 and 4
Var	3
WAMP	2 and 3
Y3	2 and 4
Y4	3 and 4
Y5	4
ZC	3 and 4

**Table 18:** Classifier performance parameters for the training stage.

	FB	EB	FD	ED	RR
Sensitivity	79.88%	78.742%	68.15%	69.72%	85.99%
Specificity	92.56%	89.328%	94.82%	95.31%	98.60%
Accuracy	90.01%	87.22%	89.5%	90.20%	96.08%
Efficiency	87.48%	85.097%	84.16%	85.08%	93.562%

**Table 19:** Classifier performance parameters for the validation stage.

	FB	EB	FD	ED	RR
Sensitivity	79.83%	78.36%	69.70%	69.57%	86.10%
Specificity	92.38%	89.42%	94.77%	95.49%	98.81%
Accuracy	89.88%	87.21%	89.75%	90.30%	96.27%
Efficiency	87.36%	84.99%	84.74%	85.12%	93.73%

## Data Availability

Not applicable.
